# Flexible Work Arrangements and Employees’ Knowledge Sharing in Post-Pandemic Era: The Roles of Workplace Loneliness and Task Interdependence

**DOI:** 10.3390/bs13020168

**Published:** 2023-02-14

**Authors:** Jin Cheng, Xin Sun, Yana Zhong, Kunlin Li

**Affiliations:** 1 School of Management, Xiamen University, Xiamen 361005, China; 2 Center for Management Philosophy and Organizational Ecosystem, Renmin University of China, Beijing 100872, China

**Keywords:** FWAs, affective events theory, workplace loneliness, knowledge sharing, task interdependence

## Abstract

Flexible work arrangements (FWAs) have become prevalent working norms in the post-pandemic era, but are they beneficial to employees’ work? From the theoretical perspective of social exchange, previous studies have viewed FWAs as supportive practices that facilitate employees’ functional intrapersonal outcomes. However, little is known about the interpersonal effects of FWAs. Based on the affective events theory, this study aims to elucidate why and when FWAs are associated with employees’ knowledge sharing. A web-based survey of 314 respondents (Study 1) and a three-wave field research study of 343 employees (Study 2) provided valid questionnaires to examine the hypothesized theoretical relationships. Our findings reveal that employees who frequently adopt FWAs would produce a persistently negative affective experience—workplace loneliness—further discouraging their intentions to share knowledge with coworkers. The specific work-characteristic conditions in this relationship–task interdependence would mitigate the dysfunctional effect of FWAs on employees’ knowledge sharing via workplace loneliness. Our study advances the understanding of FWAs’ dysfunctional impacts on employees’ knowledge sharing from the theoretical perspective of affective reactions. Our findings remind managers to avoid the interpersonal pitfalls of FWAs by increasing task interdependence among employees.

## 1. Introduction

In a knowledge-based economy, companies pay more attention to knowledge management [1]. The company’s knowledge base is the source of sustainable innovation and the basis for gaining a competitive advantage [2]. The knowledge embedded inside employees is the most original form of company knowledge, and whether the knowledge of employees can be transformed into company knowledge depends on employees’ knowledge sharing [3]. Defined as the willingness and behavior of individuals in an organization to share with others the knowledge they have acquired or created [4], knowledge sharing is an indispensable premise contributing to team creativity and organizational innovation in a flexible work context [5,6].

Previous studies have suggested that individual factors, such as values [7,8], commitments [9], personality traits [10], motivations [11,12], and team-level factors, such as interpersonal trust [13] and team cohesiveness [14,15], are effective in fostering knowledge sharing in organizations. Moreover, recent evidence indicates that companies’ supportive policies and arrangements facilitate knowledge sharing among employees [16] because they shape their daily work characteristics and determine their work statuses, providing substantial support for their knowledge sharing [17,18]. Accompanying the ongoing outbreaks of COVID-19 in the post-epidemic context, many employees frequently telecommute beyond their companies and adopt flexible schedules to balance their work tasks. Flexible work arrangements (FWAs) have become a popular human resource management policy in practice [19,20,21]. The use of FWAs has significantly changed the conditions for knowledge sharing among employees [22]. However, insufficient research has considered the effects of widely implicated FWAs on knowledge sharing.

FWAs emerged as early as the 1980s, covering forms of flexi-time and flexi-locations, and were initially designed as organizations’ supportive policies for their employees [23]. Some scholars, from the theoretical perspective of social exchange, have argued that FWAs could generate a wide range of functional outcomes [24], such as promoting employees’ affective commitment [25,26] and work satisfaction [27,28], decreasing their quitting intentions toward organizations [29]. However, a small body of research is revisiting the view that FWAs help organizations and employees obtain optimistic outcomes. As Weeks (2011) suggests, FWAs may seem like friendly, supportive plans, but actually, employees rarely benefit from these flexible practices because they are burdened with more work pressure in family life and suffer from a higher work–family imbalance [30]. Some scholars also indicate that FWAs inevitably heighten the intensity and strain of work tasks [31,32], which would undermine employees’ work efforts in the long run [33] and be detrimental to employees’ long-term work goals [34].

Besides the psychological stress and work–family conflicts, recent evidence shows that using FWAs may stimulate unpleasant affective responses among employees [34] because the use of FWAs is embedded in employees’ daily work routines and would shape the events of work, which would shock the regularity of employees’ affect patterns and elicit affective responses [35]. The effects of FWAs regarding affective aspects have been largely overlooked. Moreover, the work events shaped by FWAs include changes in work schedules and locations and stimulate changes in work-related interactions between individuals [21,36]. Despite current studies’ attention to the potential hazards of FWAs, this research primarily focuses on the intrapersonal outcomes of employees, and few studies exist regarding the interpersonal impacts of FWAs on employees. In this study, we will pay attention to the affective response triggered by FWAs.

Workplace loneliness reflects the employees’ psychological and affective experience of low-quality interpersonal relationships and unsatisfied affective needs in workplace social interactions [37,38]. According to affective event theory, sparse interpersonal contact and geographic isolation events are proximal triggers of workplace loneliness [35,39]. To be more specific, flexi-time leads to employees’ irregular working time rhythms with supervisors and colleagues, while flexi-locations isolate employees geographically from their supervisors and colleagues [25]. These work events and changes induced by FWAs’ usage impede the building of social connections between employees, which subsequently frustrates their affective affiliation. Therefore, we argue that using FWAs triggers employees’ workplace loneliness.

As Wright and Silard (2021) noted, workplace loneliness is a negative affective experience that constantly drains an individual’s psychological resources. The possible consequence for employees experiencing workplace loneliness is the avoidance of social engagement and interaction [39]. In addition, individuals who feel lonely are more reluctant to engage in risky activities [40]. However, knowledge sharing implies a transfer of ownership of knowledge, also embodied as a social activity covering at least two-party interactions [4]. Accordingly, employees will likely reduce altruistic knowledge-sharing behaviors when they feel lonely.

Moreover, the effect of workplace events on individuals’ affective reactions and social intentions and behaviors depends upon contextual characteristics [35]. In the FWA scenarios, employees are linked by various weak or strong task correlations to accomplish tasks together. Task interdependence refers to how employees depend on other team or organization members to carry out their work effectively [41]. Previous evidence has shown that task interdependence would buffer the social side effects of work flexibility [42] because it would change the interactions that influence the employees’ processes of interpreting events that occur in an organization [43,44]. Since task interdependence creates task-based connections for employees, we argue that task interdependence can mitigate the negative impacts of FWAs on employees’ workplace loneliness and knowledge sharing.

**Figure 1:**
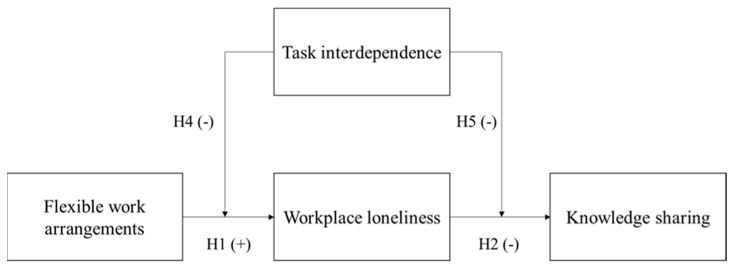
Theoretical model.

Based on affective events theory, we propose that adopting FWAs would promote employees’ affective experience of workplace loneliness, and employees who suffer from workplace loneliness are more reluctant to share their knowledge with colleagues. The dysfunctional effect of FWAs is constrained by the distinct job characteristic—task interdependence—which determines the mutual dependence of work assignments among employees under FWA scenarios [45]. Employees who select FWAs will have more workplace loneliness psychological experiences when they face a lower level of task interdependence. Our theoretical model is presented in Figure 1.

Our study makes theoretical contributions to the extant literature in several ways. First, we extended the literature on the passive effects of FWAs from an irrationally affective perspective. While previous research has discussed the impact of FWAs on individual work attitudes and behaviors based on rational perspectives such as the social exchange theory [25], our study finds that affective experiences triggered by FWAs can also impact individual behaviors. We further support Spieler et al.’s (2017) argument that the chronic implementation of FWAs can negatively affect employees’ affective states [34]. Second, we identified workplace loneliness as an affective mediating mechanism. Our study indicates that the widely implemented FWAs, especially in the post-epidemic period or a long time in the future, can stimulate employees’ perceived workplace loneliness due to the external work characteristics that employees are exposed to rather than employees’ personalities [46,47]. Third, we enriched the research on the employees’ knowledge-sharing preconditions. FWAs, as companies’ management practices, would change employees’ daily work status and further discourage the employees’ knowledge sharing. Fourth, our study clarifies task interdependence as a boundary condition to mitigate the dysfunctional effects of FWAs. Therefore, managers can alleviate the interpersonal concerns generated by FWAs by adjusting the interdependence of work tasks between subordinates.

## 2. Hypotheses Development

### 2.1. FWAs and Workplace Loneliness

FWAs, covering forms of flexi-time and flexi-locations, have been incorporated into formal human resource policies [33]. Faced with unavoidable major emergencies, such as COVID-19, many companies have to implement FWAs, which greatly subvert the employees’ traditional working way [19,20]. For example, employees work with virtual devices through the screen and communicate with their supervisors and colleagues by email, telephone, and other electronic social media. They are distributed individually in different locations and adopt variable working hours. These work events, provoked by FWAs serving as exogenous factors, shock the regularity of employees’ affect patterns, elicit affective responses, and then change employees’ relative intentions and behaviors [35].

Workplace loneliness is conceptualized as “employees’ subjective affective evaluations of, and feelings about, whether their affiliation needs are being met by the people they work with and the organization they work for” [38]. In the framework of loneliness proposed by Perlman and Peplau (1984), they identified triggering affective events as a cause of loneliness [48]. Specifically, any events disrupting employees’ social networks can be considered potential triggering events for the loneliness experience, for instance, isolation, dispersion, leaving, relocation, etc. Firstly, FWAs hinder the process of establishing intimate social relationships between employees. Social relationships are developed through various interactions, such as face-to-face and online, and these interactions create strong or weak ties. Specifically, reliable and intimate connections are based on closeness, sincerity, and camaraderie, which depend on frequent physical contact and continuous daily perception between employees [49]. In both flexi-time and flexi-location scenarios, due to inconsistent scheduling and geographic dispersion, employees tend to use virtual tools (e.g., email, Snapchat, WhatsApp, and Zoom) to interact with their supervisors and colleagues rather than traditional face-to-face communication [46]. Face-to-face communication can encompass caring signals through body language, facial expressions, and tone of voice and prompt employees’ interpersonal approachability. However, this is hard for online interactions to achieve [50,51]. In addition, flexi-time reduces the immediacy of employee communication. For employees, it is difficult to develop other positive social relationships when they are exhausted from coping with lagging messages and matters [52]. Flexi-location directly reduces the possibility of small talk in the office and unintended get-togethers after work, which are important ways to construct high-quality relationships in the workplace.

Secondly, FWAs do not meet the employees’ affective relational and belonging needs with other organizational members [53]. Each individual in the organization needs to establish high-quality relationships with others and belong to certain groups [54]. Neither flexi-time nor flexi-locations favor positive affections’ transmission, contagion, and accumulation in employees. When employees telework virtually, they cannot have physical contact with or keep an eye on their colleagues, making it difficult for them to build safe psychological climates to communicate and generate a sense of belongingness to the group [55]. In flexi-time, inconsistent schedules make each member of the group seem to be working independently and make team management seem chaotic, which is not conducive for groups to build a stable relational climate (i.e., trust, regard, and caring). More seriously, employees who keep geographically isolated and inconsistent schedules for a long time can engender feelings of alienation and disconnection from others. The psychological distress caused by a discrepancy between desired belongingness needs and low relationship fulfillment can be identified as workplace loneliness [39]. We propose the hypothesis:

**Hypothesis** **1.** *FWAs have a positive effect on workplace loneliness.*

### 2.2. Workplace Loneliness and Knowledge Sharing

In a knowledge-based economy, companies pay more attention to knowledge management [1]. The company’s knowledge base is the source of sustainable innovation and the basis for gaining a competitive advantage [2]. However, the knowledge embedded in employees is the most original form of company knowledge, and whether the knowledge of employees can be transformed into company knowledge depends on employees’ knowledge sharing [3]. Considerable evidence has shown that psychological forces, such as affective states, can drive knowledge sharing within the individual [56,57,58].

From the perspective of affect-driven behavior, workplace loneliness symbolizes an unpleasant psychological experience that is hyper-vigilant in social interaction [37]. The persistence of loneliness torments the employees and drains their energy [59]. More seriously, workplace loneliness also drives other passive emotions, such as anxiety, sadness, and depression, which lead employees to be involved in the emotionally disturbing depletion of more resources. However, knowledge sharing requires sharers to consume plenty of time and effort to transfer knowledge to recipients [59]; it is essentially an extra-role behavior and needs extra psychological resources to support, so it is hard for those suffering from loneliness to engage in such altruistic conduct. Previous studies also indicated that lonely individuals usually adopt a sad, passive strategy and appear to behave in a self-absorbed and socially ineffective manner towards others to cope with loneliness [60].

Workplace loneliness also impacts individuals’ evaluative judgment processes, especially regarding the congruence between the directions of appraisal outcome and the affective orientations, also known as the biasing effect of affect states on evaluative judgments [35]. Knowledge sharing reflects the public goods dilemma faced by the sharers. Engaging in knowledge sharing implies that the sharers are exposed to potential risks such as loss of knowledge ownership, the value of uniqueness, and expert status [61]. Therefore, the sharers need to judge the situation and assess the risks, and the intention to share will only arise when the sharers feel safe enough [62]. However, lonely individuals would interpret their situation as pessimistic, so insecure assessments about their social environment will come to mind when they judge expected reciprocity norms before sharing knowledge, which amplifies the appraised risks for knowledge sharing [40]. In addition, relational identities with colleagues, specifically the identities of the strength and the quantity of the relationship, are vital factors in generating trust, which constructs preconditions for knowledge-sharing willingness [63]. Because of the defective relational identity, the loners would not take potential risks to share knowledge with other colleagues [60]. We propose that:

**Hypothesis** **2.** *Workplace loneliness has a negative effect on knowledge sharing.*

### 2.3. The Mediating Effect of Workplace Loneliness

According to affective events theory, when work events with affective significance happen or generate changes, corresponding affective reactions follow, and employees’ affective states largely influence their subsequent intentions and behaviors [35]. A series of changes or events arising from using FWAs (flexi-time and flexi-locations) have vastly changed the relational embeddedness of employees in the workplace social structure. These affective events’ happenings and changes induced by FWAs further hinder the establishment of meaningful connections between employees and facilitate them to produce a reduced sense of personal control over social relationships [38]. When employees fail to build intimate relational connections with their supervisors and colleagues in the workplace, they feel an absence of social companionship. Moreover, these unsatisfied relationship-related needs frustrate employees’ social belongingness in the workplace, making them experience affective deprivation; these are precisely two manifestations of workplace loneliness [64]. As noted by Wright and Silard (2021), the possible consequence for employees experiencing workplace loneliness is the avoidance of social engagement and interaction [39]. However, knowledge sharing involves individuals sharing the knowledge they have acquired or created with others, embodied as a social activity covering at least two-party interactions in the workplace [4]. We argue that loners in the workplace would not readily have intentions to share knowledge with their colleagues. Combining the above, we propose:

**Hypothesis** **3.** *Workplace loneliness mediates the relationship between FWAs and knowledge sharing.*

### 2.4. The Moderating Role of Task Interdependence

Task interdependence requires each employee participating in the collective task to play their unique role and put effort into achieving the task goals, which determines the amount of interpersonal interaction under FWAs [65]. During the task process, employees initially communicate and spontaneously discuss with their colleagues, and they can gradually realize their work’s significance and worth in the team [66]. Therefore, even though the employees are geographically and temporally distant from the group in flexi-locations and flexi-time settings, they can still feel a meaningful presence in the team due to the connections created by the work tasks in a higher task interdependence context. This awareness of the self-importance of task-based links could partially replace missing social relationships caused by FWAs.

**Hypothesis** **4.** *Task interdependence moderates the positive relationship between FWAs and workplace loneliness.*

Task interdependence helps employees transfer their attention from individual tasks to collective tasks [67], and employees have fewer psychological resources to allocate to the internal depletion of workplace loneliness. A higher level of task interdependence will lead to consistent goals among employees, prompting team members to develop group cohesion and a sense of collective efficacy [68]. It enables employees to move towards a consistent working pattern, which stimulates a collaborative centripetal process and further prompts them to develop collective commitment in flexible work situations [69]. When the task is completed, employees will create a task-based sense of collective achievement and belongingness wherever and whenever they are. Subsequently, these gradually accumulating positive psychological experiences can act as an “antidote” to individuals’ passive affective states, which could partially compensate for the deprivation of affective demands caused by workplace loneliness. Employees may engage in altruistic knowledge sharing only when they have sufficient psychological resources [9].

Moreover, task interdependence can add contacts and engagements between employees under FWAs, facilitating task-focused cooperation and trust [68,70]. A high level of task interdependence implies that each person contributes information and resources to help the collective accomplishment of the task [67]. As the exchange frequency increases, the employees’ mutual understanding and trust are gradually established. Therefore, the collaboration and trust that lonely employees cannot build up can be complemented in a work setting with higher task interdependence. In addition, Ramamoorthy and Flood (2004) have indicated the mitigating effect of task interdependence on the negative relationship between solitary work preference and altruistic behavior [71]. Based on the above, as task interdependence in the work setting increases, employees’ workplace loneliness due to flexi-time and flexi-locations is alleviated, and the negative relationship between FWAs and employees’ knowledge sharing can be mitigated as well. Therefore, we hypothesize that:

**Hypothesis** **5.** *Task interdependence moderates the negative relationship between workplace loneliness and knowledge sharing.*

**Hypothesis** **6.** *Task interdependence moderates the mediating effect of workplace loneliness between FWAs and knowledge sharing.*

## 3. Study 1: A Web-Based Questionnaire Study

### 3.1. Sample and Procedure

We administered the first study’s sample group on an online platform in mainland China, which possesses functions equivalent to Amazon Mechanical Turk [72,73]. As such, the sample group for Study 1 is drawn from a diverse range of industry sectors (i.e., internet, telecommunications, biopharmaceuticals, finance, consulting services, education, and other industries). To qualify for the study, participants had to be full-time employees. To ensure that participants had a clear understanding of FWAs, we described FWAs accordingly at the beginning of the questionnaire: FWAs represent the work practices offered by the company to its staff, and specifically, employees have the flexibility to choose where they work and when they work [25,74]. Then, they were instructed to complete a web-based questionnaire containing demographic information about the participants and variables involved in our research model. In total, 383 employees completed the research questionnaire. After eliminating invalid questionnaires (e.g., incomplete completion, failure to pass test questions, presence of obvious patterns), we yielded our final sample of 314 employees. Of the samples, 47.50% were male, and the sample age was mostly (93%) in the 18- to 40-year-old range. Because older populations are insensitive to online techniques, most participants were under the age of 40, 79.30% had a bachelor’s degree or higher, 86.30% had been working for less than ten years, and 44.20% were from private enterprises and foreign enterprises.

### 3.2. Measures

The variables involved in the two studies were derived from well-established scales, and we used a back-to-back translation procedure to ensure that the scales were applicable in the Chinese context. Except for the control variables, all other variables involved in the two studies were measured using a five-point Likert-type scale.

The use of FWAs: We measured this variable using a six-item scale, which was first developed by Rau and Hyland (2002) and adapted by Shockley and Allen (2007) [74,75]. A sample statement was: ‘Over the past six months, how often have you varied your work schedule’, and the answers ranged from 1 (never) to 5 (always). The Cronbach’s α for Study 1 was 0.93, while for Study 2 it was 0.95.

Workplace loneliness: We used the 16-item scale developed by Wright (2005) to measure workplace loneliness [64]. Respondents were requested to indicate their disagreement or agreement from 1 (strongly disagree) to 5 (strongly agree). Sample items include: ‘I often feel abandoned by my co-workers when I am under pressure at work,’ and ‘I have social companionship/fellowship at work (R).’ The Cronbach’s α of Study 1 and Study 2 were 0.92 and 0.97, respectively.

Knowledge sharing: Knowledge sharing was operationalized using the five-item scale developed by Bock et al. (2005) [4]. All items were rated on a scale that ranged from 1 (strongly disagree) to 5 (strongly agree), and a sample item included: ‘I will share my work reports and official documents with members of my organization more frequently in the future.’ The Cronbach’s α was 0.86 in Study 1 and 0.87 in Study 2.

Task interdependence: Based on the measure developed by Campion et al. (1993), a three-item scale was used to assess task interdependence in this study [76]. A sample item was: ‘I cannot accomplish my tasks without information or materials from other members of my team.’ Study 1’s Cronbach α was 0.83, while Study 2’s was the same.

Control variables: As workplace loneliness and knowledge sharing are influenced by differences in individual characteristics, and in conjunction with the operations of previous research, such as Anand and Mishra (2021) and Kröll et al. (2021), both of the two studies selected respondents’ gender, age, education, tenure, and years of teamwork as the control variables [52,77].

### 3.3. Analysis Strategy

Our studies used SPSS 25.0 and Mplus 8.3 software for the statistics and analysis of the data. First, we assessed the reliability and descriptive statistics of the variables with the help of SPSS and conducted a common method bias test and confirmatory factor analysis to assess the discriminant validity of the variables with the help of Mplus. Second, we used the hierarchical regression method to examine all direct effects and the moderating effect. Finally, we further used the SPSS PROCESS procedure Bootstrap to test the mediating effect of workplace loneliness and the moderated mediation effect [78].

### 3.4. Results of Study 1

#### 3.4.1. Common Method Bias and Confirmatory Factor Analyses

We conducted the common method bias test and confirmatory factor analyses in Mplus 8.3. We added a common method variable into the four-factor model to form a five-factor model, and then compared the fit indices before and after adding this variable [79]. The results are shown in Table 1, and, with the addition of the common method latent variable, the five-factor model did not exhibit better fits than the four-factor model. This result indicated that our data were not subject to the common method bias.

Further, to evaluate the discriminant validity of our core variables, we constructed other factor models by combining some of the core variables. Specifically, the three-factor model combined FWAs and task interdependence, the two-factor model combined FWAs and workplace loneliness, task interdependence and knowledge sharing separately, and the one-factor model combined all four variables. Compared to other factor models, the four-factor model exhibited the best model fit superiority: χ^2^ = 860.53, df = 342, TLI = 0.89, CFI = 0.90, RMSEA = 0.07, SRMR = 0.06, indicating a relatively acceptable fit between the proposed model and the observed data and significantly discriminant validity among these four variables [80].

#### 3.4.2. Descriptive Statistics Analysis

Table A1 (see Appendix A) demonstrates the means, standard deviations, and correlation coefficients of each variable in Study 1. There was a significant positive correlation between FWAs and workplace loneliness (*r* = 0.26, *p* < 0.01) and a significant negative correlation between workplace loneliness and knowledge sharing (*r* = −0.41, *p* < 0.01).

#### 3.4.3. Hypothesis Testing

**Figure 2:**
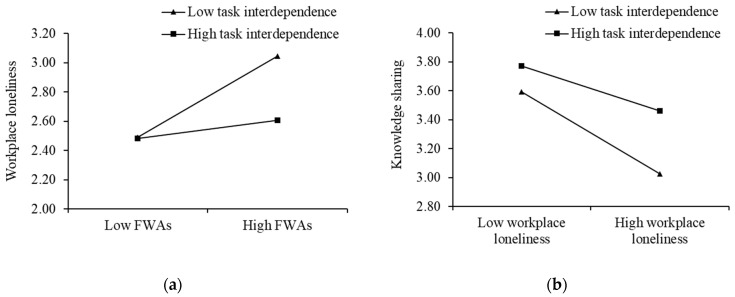
The moderating role of task interdependence in Study 1: (**a**) The moderating role of task interdependence on the relationship between FWAs and workplace loneliness; (**b**) The moderating role of task interdependence on the relationship between workplace loneliness and knowledge sharing.

We used the hierarchical regression method to test H1, H2, H4, and H5. In Table 2, the results of Model 2 (*b* = 0.18, *p* < 0.01) indicate that FWAs positively influence employees’ perceived workplace loneliness, and H1 is supported. As shown in Model 7, employees’ perceived workplace loneliness negatively influences their knowledge sharing (*b* = −0.38, *p* < 0.01), accepting H2. Before we examined the moderating effect of task interdependence, we mean-centered the FWAs, task interdependence, and workplace loneliness to calculate the interaction terms (INT), and such operations could eliminate the threats of multicollinearity. The results of Model 3 (*b* = −0.14, *p* < 0.01) and Model 8 (*b* = 0.12, *p* < 0.05) indicated the moderating role of task interdependence on FWAs and workplace loneliness, workplace loneliness and knowledge sharing, which suggest H4 and H5 are accepted. We referenced Aiken et al.’s (1991) procedure of ±1 standard deviation to plot the moderating effect, displayed explicitly in Figure 2a,b separately [81].

To further verify the mediating effect of workplace loneliness and the moderating effect of task interdependence on the indirect effect, we conducted the analyses using the Bootstrap method [63]. As in Table 3, at Bootstrap = 5000, the 95% confidence interval (95% *CI*) for the indirect effect is [−0.11, −0.03]. Therefore, the indirect effect of FWAs on knowledge sharing through workplace loneliness is significant, supporting H3. For H6, according to the results in Table 3, the confidence interval of the difference between the higher and lower levels of task interdependence is [0.01, 0.07], indicating that the moderated mediation effect is significant, supporting H6.

## 4. Study 2: A Multi-Wave Field Study

### 4.1. Participants and Procedure

The data were obtained from a large group company specializing in the information technology field in mainland China, operating in cloud computing, big data, communications, and other industries. Since the outbreak of the COVID-19 epidemic in 2020, the group company has implemented FWAs and continues to do so today. We used a three-wave questionnaire collection format to collect the data [82]. In cooperation with the human resources manager at the group headquarters, we obtained information and contact details for 800 employees from 34 departments. The questionnaire was sealed in the form of an envelope package before distribution, and the envelopes were coded.

Time 1 focused on collecting FWAs, task interdependence, and demographic variables. A custom pen with the logo of the researcher’s university, valued at approximately USD 0.80, was given inside the envelope and distributed by the companies’ HR employees. A questionnaire box was placed at the group’s headquarters, and respondents were allowed to fill out and drop their questionnaires in the box within one week. A total of 616 questionnaires were obtained for Time 1, and 518 valid questionnaires were obtained after excluding those with missing data. At Time 2, occurring two weeks after Time 1, 518 questionnaires were distributed. This round mainly collected data on employees’ perceived workplace loneliness, with a custom keyring valued at approximately USD 1.40 placed inside the envelope and distributed to the respondents. A total of 463 questionnaires were obtained for Time 2, of which 432 were valid. At Time 3, similarly, two weeks after Time 2, we distributed 432 questionnaires and placed CNY 5 in cash, approximately USD 0.8, in sealed envelopes to collect data on employees’ knowledge sharing. This round obtained a total of 378 questionnaires, with 343 valid questionnaires.

The proportion of men and women in the valid sample was relatively balanced, with women accounting for 50.70%. The sample company belongs to the high-tech industry, which develops rapidly and requires high technical expertise from staff, thus, the sample as a whole was young, with those under 35 accounting for 75.20% and those who were unmarried accounting for 63.60%. The proportion of those who had received a higher education was 43.70% for undergraduates and 33.20% for postgraduates; tenure mainly was below 15 years, accounting for 91.60%.

### 4.2. Results of Study 2

#### 4.2.1. Common Method Bias and Confirmatory Factor Analysis

The results of the common method bias test are shown in Table 4. When the common method variable was added to the four-factor model, χ^2^ decreased by 1.64 and df decreased by 1. The RMSEA, CFI, and TLI were unchanged. The results show that severe common method bias did not influence our data. Consistent with Study 1, according to Table 4, the four-factor model exhibited the best model fit superiority compared to other models: χ^2^ = 983.70, df = 342, TLI = 0.92, CFI = 0.93, RMSEA = 0.07, SRMR = 0.05, indicating significant discriminant validity among these four variables.

#### 4.2.2. Descriptive Statistics Analysis

Table A2 (see Appendix A) demonstrates the descriptive information of Study 2. As shown, there was a significant positive correlation between FWAs and workplace loneliness (*r* = 0.49, *p* < 0.01) and a significant negative correlation between workplace loneliness and knowledge sharing (*r* = −0.39, *p* < 0.01). These results all provide tentative support for H1 and H2.

#### 4.2.3. Hypothesis Testing

**Figure 3:**
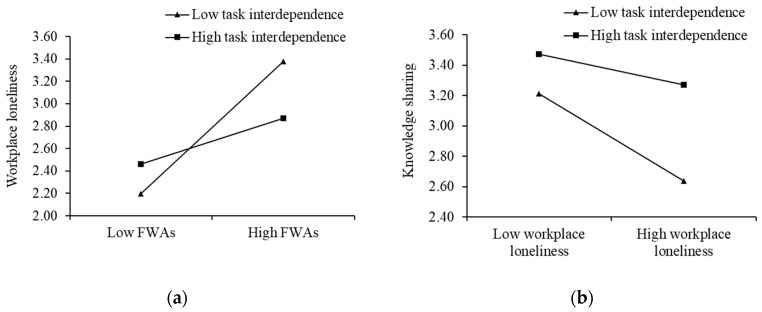
The moderating role of task interdependence in Study 2: (**a**) The moderating role of task interdependence on the relationship between FWAs and workplace loneliness; (**b**) The moderating role of task interdependence on the relationship between workplace loneliness and knowledge sharing.

According to Table 5, the unstandardized coefficient of Model 2 is 0.41 (*p* < 0.01), indicating that as employees use FWAs more frequently, the more loneliness they feel in the workplace, accepting H1. The unstandardized coefficient of Model 7 is −0.29 (*p* < 0.01), suggesting that individuals who perceive loneliness are less willing to share their knowledge, supporting H2. In addition, the results of Model 3 (*b* = −0.27, *p* < 0.01) and Model 8 (*b* = 0.16, *p* < 0.01) show that the effects of the interaction terms on workplace loneliness and knowledge sharing are both negatively significant, indicating that task interdependence weakens the positive impact of FWAs on loneliness, and also mitigates the detrimental effects of loneliness on knowledge sharing, so H4 and H5 are accepted. The simple slope plot of the moderating effect is shown in Figure 3a,b.

As for H3 and H6, we also applied the Bootstrap method through the SPSS PROCESS procedure for testing. Table 6 shows the results, and, when Bootstrap = 5000, the 95% *CI* for the indirect effect is [−0.16, −0.06], which indicates that the indirect impact of FWAs on knowledge sharing through loneliness is significant, supporting H3. In addition, the coefficient of the indirect effect of workplace loneliness at lower task interdependence is −0.10 (95% *CI* = [−0.17, −0.03]), while the coefficient of the indirect effect of workplace loneliness at higher task interdependence is −0.03 (95% *CI* = [−0.07, −0.01]), showing that high levels of task interdependence mitigate the indirect effect of FWAs on knowledge sharing, supporting H6.

## 5. Discussions

A web-based questionnaire survey (Study 1, *N* = 314) and a three-wave field survey (Study 2, *N* = 343) provided data for our theoretical model, and all proposed hypotheses were accepted.

For Hypothesis 1, the results of our two studies suggest that the extensively implemented FWAs during the post-epidemic period triggered a negative affective experience of workplace loneliness among employees. Whether flexi-time or flexi-locations, these flexible work arrangements significantly interrupt employees’ daily work routines, provoking a range of affective events and changes for employees. For example, employees work with virtual devices through the screen and communicate with their supervisors and colleagues by email, telephone, and other electronic social media. They are distributed individually in different locations and adopt various office hours when they work. In the long run, these situations are not conducive to the establishment of social relationships and the satisfaction of belonging affective needs among employees.

For Hypothesis 2 and Hypothesis 3, our results indicate that workplace loneliness mediates the negative relationship between FWAs and employees’ knowledge sharing. The affective mechanism is a crucial mediating pathway through which FWAs’ usage influences employees’ interpersonal behaviors. Specifically, in flexible working scenarios, when employees feel the perceived absence of social relationships and deprivation of affective connections at work, they decrease their participation in altruistic social activities, which avoids the further depletion of psychological resources. Meanwhile, knowledge sharing also requires a high level of interpersonal trust and requires the sharer to take the risk of losing ownership of the knowledge. Individuals who feel lonely are less likely to trust others in their social activities and less willing to take additional risks for others.

For Hypothesis 4, Hypothesis 5, and Hypothesis 6, our results reveal that employees’ affective responses towards FWAs and the inhibitory effect of FWAs on knowledge sharing are moderated by task interdependence. Task interdependence, as an essential work characteristic, is widely embedded in the employee’s task scenarios and determines how employees complete their work tasks. Even in temporally inconsistent and spatially separated work conditions, task-based relationships between employees create opportunities for employees to build social relationships. Motivated by the collective task objective, employees frequently exchange information with colleagues, which facilitates building task-based trust among employees. Thus, a high degree of task interdependence would alleviate workplace loneliness due to FWAs and mitigate the tendency to reduce knowledge sharing due to unpleasant lonely experiences.

### 5.1. Theoretical Contributions

First, we extended the literature on the disruptive effects of FWAs from an irrationally affective perspective. Our findings revealed that FWAs did not always yield good consequences, although they were designed to improve employees’ performance. Earlier studies on FWAs were primarily based on rational perspectives, such as social exchange and resource conservation, which view FWAs as signals that companies value their employees’ needs or as resource supports to their employees and argue that FWAs can bring employees positive outcomes [23,28]. Our findings found that FWAs could trigger a sensual, psychological experience and create a negative affective reflection of workplace loneliness, which drain the individuals’ psychological resources and make them less likely to engage in altruistic behaviors. We also further support Spieler’s (2017) argument that FWAs are not simple company management practices: they also carry some liabilities in that long-term (chronic) implementation of FWAs can have detrimental outcomes on organizational outcomes [34].

Second, we enriched the research on the employees’ knowledge-sharing preconditions. Although previous studies on the precursors of knowledge sharing have mainly been discussed from the individual level [8,11,12,45], our analysis takes an organizational level and found that FWAs as a company’s management policy impacted the employees’ knowledge sharing. While many scholars have pointed out that supportive company policies and practices promote the employees’ knowledge sharing [56,56,83,84], the results of our study show a different view: that FWAs as supportive company policies discourage the employees’ knowledge sharing. We argued that whether supportive company practices promote the employees’ knowledge sharing depends on the specific content of the policies and procedures and their particular impact on the employees’ daily work status.

Third, we developed workplace loneliness as an affective mediating mechanism from the perspective of the affective events theory. Our study responded to Wright and Silard’s (2021) call for attention to workplace loneliness [39]. Even though employee feelings of loneliness are prevalent, attention to workplace loneliness in the HR field is limited [38,85] and previous literature on the factors impacting employee loneliness has primarily focused on the internal aspects of individuals and ignored the role of the external environment [52]. Our study argued that the widely implemented FWAs, especially in the post-epidemic period, can lead to workplace loneliness, which is not due to employee personality traits [47], but due to the external work environment and characteristics that employees are exposed to [46]. This workplace loneliness will further negatively influence employees’ work attitudes and behaviors, damaging employee relationships in the long run and preventing regular communication and the exchange of work tasks [37,86].

Fourth, our study clarifies task interdependence as a boundary condition to mitigate the dysfunctional effects of FWAs. In the post-epidemic era, employee work paradigms have undergone disruptive changes [87]. With FWAs becoming the dominant working style, these changes in work patterns may inevitably induce alienating reactions in the employees’ psychological experiences and behaviors [88]. Shockley et al. (2021) suggested that task interdependence could amplify the positive relationship between employees’ communications (quality and frequency) and their performance, especially in the teleworking context [89]. Chong et al. (2020) demonstrated that task interdependence could mitigate daily exhaustion caused by task setbacks due to employees working remotely during COVID-19 [19]. Our study suggests that task interdependence can play a beneficial moderating role not only in the context of flexi-locations but also in the context of flexi-time.

### 5.2. Practical Implications

Our study confirmed that FWAs could impair employees’ knowledge sharing from an affective perspective. The results can enlighten managers to think critically about the design of FWAs. To reduce the harmful effects of FWAs, managers should pay more attention to the employees’ psychological needs and affective experiences and take measures to minimize the interpersonal disconnection between employees due to geographical isolation and time discontinuity.

For example, companies can hold more meetings to share activities and host more online group activities to enhance employee connections. Additionally, our research results show that higher task interdependence can weaken the adverse effects of FWAs. Therefore, when managers make task assignments for employees who adopt FWAs, they can assign tasks that require cooperation and coordination among colleagues and use the work tasks as a bridge to establish the connection between employees and increase the communication between them, thus enhancing the employees’ sense of belongingness and team task participation.

### 5.3. Limitations and Future Directions

First, although Study 2 adopted a three-wave research design to minimize common method bias, the data in Study 2 were reported by the same employees. Therefore, future studies can be improved in terms of method design. For example, the measurement of knowledge sharing can take the form of a combination of colleague and supervisor assessment, and the measurement of FWAs can use objective indicators.

Second, our study mainly discussed the impact of FWAs on the employees’ negative affective experience of workplace loneliness. However, the affective structure of individuals is characterized by complexity, multidimensionality, and instability [90,91]. Employees may have other affective responses in addition to workplace loneliness when facing the FWAs, and these different affective reactions may lead to distinct behavioral outcomes. Future studies could also explore the effects of FWAs on other affective experiences, such as anxiety and depression [92,93].

Finally, this study only discusses the moderating role that task interdependence plays in the harmful effects of FWAs. In addition to specific job characteristics, literature on affective events theory suggests that leadership and non-job factors (i.e., personal traits) may also play moderating roles in the relationship between FWAs and employees’ adverse affective reactions [94,95]. For example, supervisor support and the richness of the employees’ family life can help mitigate the negative affective experience with FWAs. These are all topics that can be discussed continuously in the future.

## 6. Conclusions

Since the outbreak of COVID-19, FWAs have been widely adopted by a growing number of companies. However, the mechanisms between FWAs and employees’ behaviors and performances have not been fully discussed, especially regarding employees’ affective reactions. Based on affective events theory, we find that the widely implemented FWAs induced employees’ affective responses, which are manifested as workplace loneliness, and employees who suffer from workplace loneliness are more reluctant to share their knowledge with their colleagues. In the long-term, FWAs are unfavorable for employees to develop intimate social relationships with others and are not beneficial for the company to cultivate a vibrant climate of knowledge sharing. However, our study found that increasing the interdependence of work tasks among employees can mitigate the damaging effects of FWAs on employees’ knowledge sharing. Therefore, to alleviate employees’ negative affective experiences of loneliness and their reluctance to share knowledge, managers can increase the work connections between employees when they assign tasks.

## Figures and Tables

**Table 1:** Confirmatory factor analyses of Study 1.

Factor Models	χ^2^	df	TLI	CFI	RMSEA	SRMR
One-factor model: combined four variables	3427.96	350	0.38	0.43	0.17	0.16
Two-factor model: combined FWAs and workplace loneliness, knowledge sharing and task interdependence, respectively	2846.26	349	0.50	0.54	0.15	0.15
Three-factor model: combined FWAs and task interdependence	1253.80	345	0.82	0.83	0.09	0.09
Four-factor model: no variables were combined	860.53	342	0.89	0.90	0.07	0.06
Five-factor model: added a common method variable	860.37	341	0.89	0.90	0.07	0.06

Notes: *N* = 314; χ^2^ = Chi-square; df = degree of freedom; CFI = comparative fit index; TLI = Tucker–Lewis index; RMSEA = root mean square error of approximation; SRMR = standard root mean-square residual.

**Table 2:** Hierarchical regression analyses of Study 1.

	Workplace Loneliness			Knowledge Sharing				
Models	Model 1	Model 2	Model 3	Model 4	Model 5	Model 6	Model 7	Model 8
Constants	2.85 **	2.32 **	2.72 **	3.29 **	3.79 **	4.58 **	4.37 **	3.68 **
Gen	−0.13	−0.08	−0.05	0.02	−0.02	−0.05	−0.03	−0.07
Age	0.10	0.12	0.08	−0.04	−0.06	−0.02	0.00	0.02
Edu	0.01	0.00	0.01	−0.02	−0.01	−0.01	−0.02	−0.03
Soe	−0.10	−0.01	0.01	0.33 **	0.25 *	0.24 *	0.30 **	0.25 *
Foe	0.11	0.05	0.01	−0.51 **	−0.45 **	−0.43 **	−0.47 **	−0.42 **
Gov	−0.11	−0.01	−0.02	0.14	0.05	0.04	0.10	0.13
Oth	0.00	0.00	0.02	0.02	0.01	0.01	0.02	0.03
Tene	−0.03	−0.04	−0.02	0.00	0.01	0.00	−0.01	−0.02
Team	−0.11 *	−0.11 *	−0.10	0.14 **	0.13 **	0.10 *	0.09 *	0.09 *
FWA		0.18 **	0.18 **		−0.18 **	−0.11 **		
WL						−0.34 **	−0.38 **	−0.32 **
TI			−0.14 **					0.20 **
INT1			−0.14 **					
INT2								0.12 *
R^2^	0.04	0.10	0.15	0.10	0.16	0.27	0.24	0.31
ΔR^2^	0.04	0.06	0.06	0.10	0.13	0.24	0.14	0.06

*N* = 314; * *p* < 0.05; ** *p* < 0.01; INT1 = the interaction term of mean-centered FWAs and task interdependence; INT2 = the interaction term of mean-centered workplace loneliness and task interdependence.

**Table 3:** Mediation effect and moderated mediation analyses of Study 1.

	**Effect**	**SE**	**LLCI**	**ULCI**
Total effect (FWAs on knowledge sharing)	−0.18	0.04	−0.25	−0.10
Direct effect (FWAs on knowledge sharing)	−0.11	0.04	−0.19	−0.04
	Effect	BootSE	BootLLCI	BootULCI
Indirect effect (FWAs on knowledge sharing via workplace loneliness)	−0.06	0.02	−0.11	−0.03
	**Effect/Index**	**BootSE**	**BootLLCI**	**BootULCI**
Lower task interdependence (−1 SD)	−0.08	0.02	−0.13	−0.04
Middle task interdependence	−0.05	0.02	−0.08	−0.02
Higher task interdependence (+1 SD)	−0.02	0.02	−0.05	0.01
Difference	0.04	0.01	0.01	0.07

*N* = 314; Bootstrap = 5000; 95% confidence interval; SE = standard error.

**Table 4:** Confirmatory factor analyses of Study 2.

Factor Models	χ^2^	df	TLI	CFI	RMSEA	SRMR
One-factor model: combined four variables	4147.18	350	0.54	0.57	0.18	0.15
Two-factor model: combined FWAs and workplace loneliness, knowledge sharing and task interdependence, respectively	3356.12	349	0.63	0.67	0.16	0.13
Three-factor model: combined FWAs and task interdependence	1422.62	345	0.87	0.88	0.10	0.09
Four-factor model: no variables were combined	983.70	342	0.92	0.93	0.07	0.06
Five-factor model: added a common method variable	982.06	341	0.92	0.93	0.07	0.05

*N* = 343.

**Table 5:** Hierarchical regression analyses of Study 2.

	Workplace Loneliness			Knowledge Sharing				
Models	Model 1	Model 2	Model 3	Model 4	Model 5	Model 6	Model 7	Model 8
Gen	2.72 **	1.14 *	1.57 **	3.08 **	3.69 **	3.99 **	3.87 **	2.61 **
Age	−0.12	0.03	0.03	0.03	−0.03	−0.02	−0.01	0.01
Edu	−0.07	0.00	−0.01	0.07	0.04	0.04	0.05	0.06
Tene	−0.05	0.03	0.02	−0.07	−0.10 *	−0.09 *	−0.08	−0.07
Posi	0.09 *	0.05	0.05	−0.07 **	−0.06 *	−0.05	−0.05	−0.05
Sin	0.12	0.04	−0.03	−0.01	0.03	0.04	0.03	0.08
Mar	0.04	0.15	0.25	0.25	0.21	0.25	0.26	0.12
Div	0.03	0.32	0.38	0.14	0.04	0.12	0.15	0.10
FWAs		0.41 **	0.38 **		−0.16 **	−0.05		
WL						−0.26 **	−0.29 **	−0.22 **
TI			−0.09					0.33 **
INT1			−0.27 **					
INT2								0.16 **
R^2^	0.03	0.25	0.31	0.06	0.12	0.20	0.20	0.36
ΔR^2^	0.03	0.22	0.06	0.06	0.05	0.09	0.14	0.16

*N* = 343; * *p* < 0.05; ** *p* < 0.01; INT1 = the interaction term of mean-centered FWAs and task interdependence; INT2 = the interaction term of mean-centered workplace loneliness and task interdependence.

**Table 6:** Mediation effect and moderated mediation analyses of Study 2.

	**Effect**	**SE**	**LLCI**	**ULCI**
Total effect (FWAs on knowledge sharing)	−0.16	0.04	−0.23	−0.09
Direct effect (FWAs on knowledge sharing)	−0.05	0.04	−0.12	0.03
	Effect	BootSE	BootLLCI	BootULCI
Indirect effect (FWAs on knowledge sharing via workplace loneliness)	−0.11	0.03	−0.16	−0.06
	**Effect/Index**	**BootSE**	**BootLLCI**	**BootULCI**
Lower task interdependence (−1 SD)	−0.10	0.03	−0.17	−0.03
Middle task interdependence	−0.07	0.02	−0.11	−0.02
Higher task interdependence (+1 SD)	−0.03	0.02	−0.07	−0.01
Difference	0.05	0.02	0.02	0.09

*N* = 343; Bootstrap = 5000; 95% confidence interval; SE = standard error.

## Data Availability

The data and models used during the study are available from the corresponding author by request.

## Appendix A

Table A1 and Table A2 depict the means, standard deviations, and correlation coefficients between the variables for the Study 1 and Study 2 sample groups, respectively.

**Table A1:** Summary statistics and correlations of Study 1.

Variables	1	2	3	4	5	6	7	8	9	10	11	12	13	14
1. Gen	1													
2. Age	−0.15 **	1												
3. Edu	−0.05	0.14 **	1											
4. Tene	−0.11 **	0.81 **	−0.09	1										
5. Team	−0.09	0.63 **	−0.02	0.66 **	1									
6. Soe	−0.11 *	0.08	0.00	0.08	0.06	1								
7. Foe	0.10 *	0.06	0.08	0.06	0.13 **	−0.11 *	1							
8. Poe	−0.18 **	−0.01	0.01	−0.03	−0.09 *	−0.34 **	−0.20 **	1						
9. Gov	0.11 **	0.14 **	0.09 *	0.11 *	0.09 *	−0.21 **	−0.12 **	−0.39 **	1					
10. Oth	0.15 **	−0.23 **	−0.15 **	−0.18 **	−0.11 *	−0.22 **	−0.12 **	−0.40 **	−0.25 **	1				
11. FWAs	−0.13 **	0.01	0.05	0.03	0.01	−0.12 **	0.13 **	0.16 **	−0.19 **	0.03	1			
12. WL	−0.09	0.01	0.05	−0.04	−0.10 *	−0.04	0.03	0.07	−0.07	0.00	0.26 **	1		
13. KS	−0.02	0.07	−0.05	0.09 *	0.15 **	0.19 **	−0.19 **	−0.09	0.07	−0.03	−0.29 **	−0.41 **	1	
14. TI	0.12 **	−0.04	0.12 **	0.01	0.01	0.11 **	−0.03	−0.01	−0.08	0.01	−0.11 **	−0.20 **	0.31 **	1
Mean	1.53	2.88	3.89	2.98	2.03	0.16	0.06	0.39	0.19	0.20	2.62	2.67	3.45	3.27
SD	0.50	1.00	0.89	1.59	0.99	0.37	0.23	0.49	0.40	0.40	0.95	0.69	0.67	0.79

*N* = 314; * *p* < 0.05; ** *p* < 0.01; Gen = gender; Edu = education; Tene = tenure; Team = team tenure; Soe = state-owned enterprises; Foe = foreign-owned enterprises; Poe = private-owned enterprises; Gov = government and public institutions; Oth = other organizations; WL = workplace loneliness; TI = task interdependence; KS = knowledge sharing.

**Table A2:** Summary statistics and correlations of Study 2.

Variables	1	2	3	4	5	6	7	8	9	10	11	12
1. Gen	1											
2. Age	−0.26 **	1										
3. Edu	−0.07	−0.31 **	1									
4. Tene	−0.11 *	0.36 **	0.02	1								
5. Posi	−0.14 **	0.18 **	0.14 *	0.28 **	1							
6. Sin	0.15 **	−0.75 **	0.26 **	−0.26 **	−0.17 **	1						
7. Mar	−0.13 *	0.63 **	−0.18 **	0.23 **	0.19 **	−0.93 **	1					
8. Div	−0.07	0.36 **	−0.22 **	0.10	−0.05	−0.25 **	−0.13 *	1				
9. FWAs	−0.15 **	0.05	−0.09	0.15 **	0.13 *	−0.12 *	0.10	0.05	1			
10. WL	−0.06	−0.03	0.01	0.13 *	0.10	0.02	−0.01	−0.03	0.49 **	1		
11. KS	0.00	0.15 **	−0.14 **	−0.10	−0.02	−0.15 **	0.15 **	0.01	−0.23 **	−0.39 **	1	
12. TI	−0.02	0.01	−0.06	−0.03	−0.08	−0.06	0.09	−0.08	−0.01	−0.12 *	0.41 **	1
Mean	0.51	3.91	2.36	3.30	1.22	0.64	0.33	0.04	2.58	2.73	3.14	3.05
SE	0.50	1.52	0.83	1.53	0.54	0.48	0.47	0.18	1.06	0.89	0.69	0.67

*N* = 343; * *p* < 0.05; ** *p* < 0.01; Gen = gender; Sin = single; Mar = married; Div = divorced; Edu = education; Tene = tenure; Pos = position; FWAs = flexible work arrangements; WL = workplace loneliness; TI = task interdependence; KS = knowledge sharing.
